# Stakeholder perspectives on a door-to-door intervention to increase community engagement for malaria elimination in Zanzibar

**DOI:** 10.1186/s12936-023-04474-w

**Published:** 2023-02-11

**Authors:** Faiza Abbas, April Monroe, Samson Kiware, Mwinyi Khamis, Naomi Serbantez, Abdul- Wahid Al- Mafazy, Fauzia Mohamed, Emmanuel Kigadye

**Affiliations:** 1 Zanzibar Malaria Elimination Programme, Zanzibar, Tanzania; 2 Open University of Tanzania, Dar es Salaam, Tanzania; 3 Johns Hopkins Center for Communication Programs, Baltimore, MD USA; 4 Ifakara Health Institute, Dar es Salaam, United Republic of Tanzania; 5 U.S. President’s Malaria Initiative, U.S. Agency for International Development, Dar es Salaam, Tanzania; 6 RTI International, Dar es Salaam, United Republic of Tanzania; 7 Pan African Mosquito Control Association (PAMCA), Nairobi, Kenya

**Keywords:** Door-to-door intervention, Community engagement, Community health volunteers, Local malaria transmission, Malaria incidence, Zanzibar, Intervention

## Abstract

**Background:**

Malaria remains a major public health problem in sub-Saharan Africa. The 2021 World Health Organization (WHO) World Malaria Report indicates a slowing in the decline of malaria incidence since 2015. Malaria prevalence in Zanzibar has been maintained at less than 1% since 2010, however from 2018 to 2021, the annual number of reported malaria cases has gradually increased from 4106 to 9290. Community engagement has been emphasized by the WHO for reducing malaria transmission. To better understand the potential for a door-to-door approach for malaria, a three-month pilot programme was carried out. This qualitative study aimed at understanding stakeholder experiences with the pilot programme and considerations for its implementation.

**Methods:**

Through multistage sampling, four shehias (wards—the lowest administrative structure) with comparatively high (> 1.9 per 1000) and four with low (< 1 per 1000) incidence of local malaria cases were selected and involved in a door-to-door pilot intervention. The qualitative study was conducted after the pilot intervention and employed focus group discussions and in-depth interviews. All field notes were written on paper and audiotaped using digital audio-recorders. Summaries were developed by integrating field notes with reviews of recordings; themes were developed based on the topics identified a priori. Responses for each theme were summarized using an iterative process.

**Results:**

Most community members reported high levels of acceptance of door-to-door interventions. Some factors that might affect implementation of door-to-door include, low risk perception of the disease, local beliefs and practice, lack of initiative from the programme level to involve communities, and political instability during the election period. All Community Health Volunteers (CHVs) recommended this approach for community engagement, however, ensuring adequate resources was identified as a key factor for ensuring its sustainability.

**Conclusion:**

The door-to-door intervention was perceived as helpful for promoting community engagement. There are several factors to consider including ensuring that CHVs are provided with adequate education, regular supervision, and have access to essential resources. Community leaders should be fully involved in choosing CHVs that are acceptable to the community. To ensure sustainability, the government should allocate sufficient resources and improve coordination systems.

## Background

Malaria remains a major public health problem in sub-Saharan Africa, including Tanzania. The 2021 World Health Organization (WHO) World Malaria Report indicates a slowing in the decline of malaria incidence since 2015, with an estimated 241 million malaria cases and 627,000 deaths worldwide. The WHO African Region accounted for 228 million cases (95%) of all reported cases [1].

In Zanzibar, the prevalence of asymptomatic infection in the general population has declined from above 25% in 2005 to less than 1%, where it has been maintained since 2008 [2, 3]. Despite these achievements, progress has begun to stall even while the coverage of malaria interventions across the island continues to be high. From 2018 to 2021, the annual number of reported malaria cases has gradually increased from 4106 to 9290 [4, 5]. The annual parasite incidence increased from 2.7 per 1000 populations in the strategic plan baseline (2017) to 4.06 in 2019 [6].

In light of stalled progress, a shift towards creating local and site-specific solutions is needed, and effective community engagement must be an essential component of the response. The WHO has defined community engagement as “a process of developing relationships that enable stakeholders to work together to address health-related issues and promote well-being to achieve positive health impact and outcomes” [7].

The WHO framework for malaria elimination has emphasized the importance of community engagement in eliminating malaria, especially in areas with very low malaria prevalence. The required level of intervention coverage, particularly as malaria prevalence is reduced to very low levels, can be achieved, and sustained only if communities are fully supportive [8]. The success of malaria prevention and case management efforts depend heavily on individual and community-level behaviours from consistent use of prevention measures to prompt care-seeking for fever [9, 10].

Community Health Volunteers (CHVs) provide several advantages and can contribute significantly to the provision of health education. In Zanzibar, just like in many other countries, the CHVs are from and live closer to the community that they visit, they have more understanding of the environment and the problems the community faces. With this consideration, they may find it easier to communicate with the community and gain trust from their patients. A few studies have documented that CHVs can enhance the cultural relevance of health materials and information; they may be able to shape the healthcare system to suit their community needs and can be cost-effective extensions of the health system [11–13]. Thus, CHVs are often considered a critical link between communities and the formal health system. They are seen as a means to ‘reach the last mile’ when implementing programmes, removing barriers to healthcare within the community [14–16].

One approach used to reach community members is a door-to-door approach. In this approach, trained community or health volunteers visit each house in a community and provide tailored health education or services. While few studies have evaluated the impact of door-to-door campaigns, available evidence suggests they have potential to increase utilization of malaria interventions [17]. Hang-up campaigns carried out by community health volunteers following mass campaigns or prior to peak transmission periods to ensure that insecticide-treated nets (ITNs) are hanging and used in each house are not recommended for programmatic implementation due to cost involved. However, in one study, a door-to-door hang-up approach was used following long-lasting insecticidal net (LLIN) campaigns, which involved door-to-door visits of households with educational messages. It was found that households that received this intervention, particularly, the most recent intervention visit, had higher levels of net use than the control households [17].

Another door-to-door approach was piloted in Senegal for the provision of Seasonal Malaria Chemoprevention (SMC), which is recommended by the WHO for children who live in areas with intense and highly seasonal malaria transmission. The results suggested that a door-to-door approach was more effective than delivery through fixed points (common selected community spaces including health centres, churches and schools) [18].

In Zanzibar, the Jamii ni Afya (Family is Health) programme was officially launched in February 2020 by the Ministry of Health under the Health Promotion Department and partners, when Zanzibar’s National Community Health Strategy (ZCHS) 2020–2025 was released. In this strategy, CHVs are recognized as an essential cadre to expand access to universal health coverage, reaching every household in Zanzibar (approximately 253,608 households, according to the Office of the Chief Government Statistician report of June 2021) [19]. The key roles of CHVs are delivering integrated, comprehensive reproductive, maternal, newborn, child health and child development services. Jamii ni Afya uses digital platforms to inform programme management and quality improvement. Data on visit length, location, and content can support CHV supervision, mentorship, workforce management, and resource allocation [20].

In the Zanzibar Community health strategy, local leaders have been identified as key stakeholders. Implementing partners rely on either the health facility in-charge or sheha (local leaders) to identify potential candidates to serve as CHVs. The criteria used to select CHVs have been highlighted in this strategy, some of the criteria are: age between 20 and 55, being a resident in the respective community they will be working in, an education background with a minimum ability to read and write, willingness and readiness to work as a volunteer, and being acceptable and respected by the respective community [21]. A clear identification and selection process of CHVs from announcement to selection has also been documented emphasizing ownership of the community leaders and community health committee in the overall process of identification, selection and supervision of CHVs activities living in their *shehia* (wards/lowest administrative structure). CHVs in Zanzibar are provided with a standardized package of financial, non-financial and social incentive [21].

Currently, there are about 2300 CHVs in Zanzibar distributed in all districts reaching the household with pregnant mothers and children under 5 years of age only. An additional set of intervention packages for CHVs was developed in 2021 on HIV, TB, NTDS, Nutrition and Malaria but have not yet been implemented.

To better understand the potential for a door-to-door approach for the malaria intervention, a three-month pilot programme was carried out. The results of a qualitative study aimed at understanding stakeholder experiences with the pilot programme and considerations for its implementation, are presented here. Experience gained from this pilot will help inform and strengthen CHV services and the rollout of expanded malaria services by CHVs in Zanzibar and other similar settings in Africa.

## Methods

### Study area

Zanzibar is the Tanzanian archipelago located 25–50 kms off the east coast of the Tanzania mainland in the Indian Ocean. The climate is tropical, hot all year round with two peak periods of malaria transmission associated with seasonal rainfall patterns. The main peak being May to June and low peak in October to November. Zanzibar is made up of 5 regions, 11 districts, and 332 Shehia. This study was conducted in two Unguja districts which are Kusini and Kaskazini B.

### Sampling design

Through multistage sampling, eight shehias were selected by the researcher to participate in a pilot programme based on incidence of locally acquired, confirmed malaria: four shehias were grouped with an incidence ≥ 1.9 per 1000 population and four shehias were grouped with an incidence < 1 per 1000 population.

### Pilot description

The researcher carried out a three-month pilot study from June–September 2021 and involved 55 CHVs, selected with the help of community leaders, with an average of 145 houses served by each CHV. CHVs received five days of training and checklist to guide provision of health education. Topics included the malaria situation in Zanzibar, causes of malaria, symptoms of malaria, prevention of malaria, early health-seeking behaviour and malaria elimination interventions including surveillance activities. The CHVs visited each household twice a month for three months and they were incentivized with 45,000 Tshs ($19) a month on top of the training allowances and other incentives like working tools.

### Data collection

A qualitative descriptive study was carried out to collect in-depth information on the door-to-door pilot intervention just after it was completed, between October and December 2021. The qualitative study employed Focus Group Discussions (FGDs) and In-depth Interviews (IDIs) to better understand the experiences of community health volunteers, community members, local leaders, and stakeholders at different levels within the Ministry of Health with the intervention. Data was collected by trained research assistants who were experienced members of the District Health Management teams.

Semi-structured IDI and FGD guides were designed in English and translated into Swahili (local language spoken by all Zanzibaris). Topics included in discussion guides are included in Table 1. Research assistants who were proficient in Swahili, the local language, were recruited and trained by the researcher.

**Table 1:** IDI and FGD guide topics

In-depth interview	Applicability and acceptability of door-to-door intervention Factors that may affect implementation Existence of policy to support implementation Experience working with community health volunteers in the previous programmes Availability of resources to implement this intervention
FGD with community members	Involvement in planning and implementation of malaria interventions in community Acceptance of intervention by general public Barriers to implementing intervention at community level Perceptions of door-to-door as a strategy to increase community engagement for malaria elimination
FGD with CHVs	Perceptions of door-to-door as an approach to increase community engagement in malaria elimination efforts Proposed working conditions if approach is adopted Experience and lessons learned during door-to-door intervention

#### Focus group discussions

Focus group discussions were conducted with 10 community members for each group; due to cultural consideration male and female FGDs were conducted separately. The aim of FGDs was to collect normative views on community engagement using door-to-door approach from both men and women living in communities that received the intervention. Community leaders assisted in the identification of focus group participants who were ≥ 18 years of age and willing to participate. FGDs were also conducted with community health volunteers involved in the study to explore their experience in the provision of door-to-door health education on malaria elimination interventions. The FGDs were completed within 60–80 min. FGDs were conducted in Swahili, all field notes were written on paper and discussions were audiotaped using digital audio-recorders; translation was done by the research assistants after the FGDs ended.

#### In-depth interviews

All IDIs were conducted in Swahili and focused on assessing how applicable the door-to-door approach is for accelerating malaria elimination. Participants were purposively selected based on their roles and experience in implementing community health interventions (Programme staff, District health management teams, Health care providers and community leaders). The IDIs were completed within 40–60 min.

### Data analysis

All field notes were written on paper and discussions were audiotaped using digital audio-recorders. Audiotaped data were transcribed into Microsoft Word for Windows, and later translated into English by researchers. Weekly review meetings were conducted with field staff to track progress and resolve any challenges observed during the data collection. Recordings and transcripts were reviewed by both field staff and the researchers to ensure quality. Summaries were developed by integrating field notes with reviews of recordings. A thematic approach to data analysis was used. Themes were developed based on the topics identified a priori and refined through review of the data. Responses for each theme were summarized using an iterative process, and the data were manually analyzed.

## Results

A total of 32 IDIs were completed with national level programme managers and Social Behavioural Change (SBC) staff from (Malaria, Integrated Reproductive and Child Health (IRCH), Zanzibar Integrated Hepatitis, HIV, TB and Leprosy Programme (ZIHHTLP), Nutrition unit, Health Promotion, Neglected Tropical Diseases and non-Communicable diseases), health care providers, District Health Management Teams, and community leaders. A breakdown of IDIs per category is provided in Table 2. 16 FGDs were conducted with 160 community members across the eight shehias. One additional focus group was carried out with 12 randomly selected CHVs to gather more information on their experience with the door-to-door campaign and allow for rich discussion across participants. Results are organized around key topics of interest.

**Table 2:** No of respondents for IDI

No	Type of respondents	Number of respondents
1	Programme managers and (SBC) staff at national level	14
2	Health care providers (health facility in charges)	8
3	District health management team (health directors)	2
4	Community leaders (Sheha)	8
	Total	32

The majority of respondents reported receiving information on malaria prevention from different media including television and radio and sometimes get an opportunity to ask questions through live radio and TV programmes. A few people had someone in their family test positive at the health facilities, hence were visited by District Malaria Surveillance Officers (DMSOs) for health education, household investigation and malaria testing of other household members, others were not visited as there was no one in their family who got sick with malaria.

### Factors affecting implementation of community engagement using door-to-door in provision of health-related services

Programme staff, District Medical Officers, health care providers and community leaders provided insights on factors that might affect implementation of community engagement using door-to-door based on their experience with community members.

These included factors related to governance and management, such as lack of skills and initiative from the programme level to promote participatory methodology and techniques; lack of data use for decision making at the health facility to identify priority interventions at the community level; low levels of involvement of health care providers in planning interventions with most planned at the programme level and sent to health care providers for implementation; and lack of linkage between programmes, health care providers and communities.

“Design and implementation of the door-to-door intervention will require enforcement in implementation of the community health strategy and capacity building of National, district and health facility staff on participatory approaches and community engagement”. (Programme staff).

One health care provider added:

“Although we are working close to the community we are serving, most of the time we have not been involved in planning the interventions for our community, which reduces our sense of ownership and inhibits sustainability and proper follow up of these interventions at the community level”.

Community members during the FGDs also described social, political, and economic factors that could impact implementation. This included gender norms and practices such as the perception that health issues are only concerning women; limited resources to fund the activities at the community level; and political barriers such as unwillingness to take part in community activities if the community leader is from the opposition party, and political instability during the election period. Participants also mentioned that low risk perception of the disease among community members, level of education, and focus on meeting daily subsistence needs among low-income community members could all impact engagement.

### Experiences with door-to-door intervention

Many community members reported being interested in the door-to-door intervention and gaining more knowledge from the education they received through community health volunteers.

“It is more than five years since a member of this family was confirmed with malaria, we heard that this disease is no longer in Zanzibar, and we stopped protecting ourselves. We are grateful for the education we got from CHVs and now we understand that even with the low transmission there is still risk of getting malaria and we need to continue protecting ourselves and keeping our environment clean”. (Female, community member, kijini).

Another participant argued:

“The Government is putting a lot of effort in eradicating malaria. My neighbor was visited by health care providers for household testing of malaria once their child was sick, I suggest this (door-to-door) intervention for each household because we all need this education to continue protecting our families rather than being visited when a household member is (already) sick”.—(Female, Community Member, Bwejuu).

Participants requested for this service to continue, reporting it changed their understanding of malaria and made them feel they have a role to play in helping the government fight the disease. They recommended that the CHVs should be identified from their locality, should be recognized by community leaders and health providers and should adhere to cultural norms of that particular community.

“We would like to continue receiving health education through home visits by our fellow community members who have received special training, we are happy and feel comfortable receiving these services from the people who are accepted in our own community”. —(male, Community Member, Mafufuni).

These findings were echoed by CHVs who reported that many people were happy that they reached out to them and provided them with this education.

“Community members were asking us many questions and after realizing the importance of sleeping under the insecticide treated nets, they started asking for them. During the initial first visits people were not using nets, thinking that malaria was eliminated in Zanzibar. After educating them that malaria still persists, although at a low level, and still remains a dangerous disease, many of them were encouraged to start using mosquito nets again and to keep their environment clean”.—Community Health Volunteer.

CHVs recommended the door-to-door approach for community engagement, noting that the one-on-one discussion was helpful for addressing specific challenges and concerns within a particular household and community. One CHV provided the example of helping community members obtain needed ITN, explaining, “we noticed many people were in need of ITNs and we were able to help them get them from the Sheha (community leader) through the continuous distribution modality.”

### Challenges and recommendations for implementing door-to-door intervention

While CHVs overwhelmingly supported use of the door-to-door approach, they also shared challenges. Gender norms were reported to influence the experience of community members and CHVs in the door-to-door approach. A male CHV shared, “for some of us sometimes it becomes difficult, and it can be a bit uncomfortable for the male CHV to visit and educate the pregnant women”. Due to work outside the home, male household members were often away during the door-to-door visits and were reported to be less likely to receive the intervention. During the pilot programme, two female CHVs quit, and reported the reason to be a lack of support from their husband. Participants described the importance of clear communication to households from community leaders, the need for CHVs to respect community norms including wearing acceptable clothes, respecting the time of home visits, and using the procedure to be identified by the community leader before starting the work.

To improve their work, CHVs also recommended additional training and support to help them carry out their work more effectively. Suggestions included educational leaflets to be provided to community members after the provision of health education, on-the-job training, supportive supervision, and improved working conditions. They also recommended CHVs receive additional payment for their work to enable them to attain their basic needs.

Currently due to political stability, many respondents mentioned that they do not anticipate any political barriers however during the election period the case might be different as previous experience shows that political opposition affected uptake of public services.

Many interviewees at the National Programme admitted that in many of the programmes they did not have enough resources to sustain community interventions, although they do not need large investments, equipment such as bicycles can be very helpful in reaching the community.

The Ministry of health in collaboration with implementing partners is supporting a number of community-based interventions as part of the implementation of the ZCHS. Currently all CHVs interventions are being implemented under Jamii ni Afya programme that have not been expanded to include provision of other services like malaria, the interventions are supported by D-Tree International, UNICEF and Save the Children respectively. Participants shared that shehia leadership should be on the front line in the implementation of door-to-door interventions, however they need to be guided and supported by the national programmes and the Ministry of health.

### Involvement of community members in planning and implementation of malaria and other health related interventions

Across focus group discussions, the majority of respondents reported not having been involved in the planning and implementation of malaria elimination activities. In the two high transmission shehias, they reported not to have been involved in planning, however, they were informed by the community leaders of the indoor residual spraying (IRS) and were involved in implementation. These shehias were involved in targeted IRS that was conducted in 2021. A community member from one of the high transmissions shehias shared:

“We are not involved in planning the IRS exercise, we are informed late or on the same day when the operation is taking place causing a lot of inconvenience…If we were involved in planning or informed in advance, community members could have organized themselves much better to avoid delays in preparing the houses and the coverage rate of house spray would have been at 100%.” (Male, Community member, Upenja).

## Discussion

The majority of respondents in this study reported not having been involved in the planning and implementation of malaria elimination activities with the exception of the two high transmission shehia who were informed by the community leaders of the Indoor Residual Spraying (IRS) and involved in implementation.

The WHO framework for malaria elimination emphasizes the importance of community engagement toward malaria elimination [8]. Community engagement has been defined by WHO as a process of developing relationships that enable stakeholders to work together to address health-related issues and promote well-being to achieve positive health impact [22]. There are four approaches to community engagement which are inform, consult, involve, collaborate, and empower [7]. If communities feel that they “own” programmes and are actively involved in their implementation, activities will be easier to implement, and coverage targets will more likely be reached [8].

In this study, nearly all respondents reported high levels of acceptance of door-to-door interventions as a measure to increase community engagement and felt they had learned more about malaria when they were reached by CHV staff than when they heard information on radio and television broadcasts. Many realized the importance of continuing to protect themselves from malaria even when the transmission was low.

The community recommended several considerations when providing door-to-door interventions. CHVs should respect the culture, customs, and traditions of the areas where they work, including wearing acceptable clothing and identifying appropriate times for home visits. Other studies have also emphasized the need for health service providers to consider the cultural aspects of the community while providing health related services in the community. Several systematic reviews described how indigenous people in many contexts hold a holistic view of health incorporating community, environment, spiritual, emotional, and physical well-being. Moreover, local cultural values, customs and beliefs were at the centre of and underpinned all aspects of care in indigenous service delivery models [23–25]. Although holistic view of the indigenous people in this study was not explored, additional research on this topic could be useful to help inform the interventions.

Ensuring provision of culturally sensitive, evidence-based interventions to improve health is, therefore, an important approach and has been recommended by various studies [26]. Studies have also documented how gender perspectives can affect the provision of health services in the community. Studies conducted in Nigeria and Somalia suggested that female CHWs are more accepted and assumed to be in a better positioned than male CHWs to achieve improved health outcomes for women and children. For example, the lack of acceptance of male lay health workers by pregnant women was reported to contribute to their low impact on maternal health [27]. A study conducted in Morogoro region in Tanzania found that male and female CHWs had largely similar knowledge and health promotion outputs, but challenges in acceptance of CHW counseling for reproductive health and home visits by unaccompanied CHWs varied by gender [28]. Programmes that pair male and female CHWs may potentially overcome gender issues in CHW acceptance [28]. Finally, female CHVs recommended increasing engagement with spouses during recruitment of CHVs to ensure buy-in and support for their work to prevent drop off of CHVs over time. This recommendation is likely linked to social and religious norms in Zanzibar, and the practice may differ in other places. Gender considerations for CHVs have also been reported in other studies. Notably, socio-cultural norms that restrict the movement of female CHWs and govern acceptable male–female communications have been identified as a barrier to doing their jobs successfully [14, 29, 30].

Although the majority of these studies focused on the provision of services for reproductive and child health, the same group is considered to be at most risk of malaria in many areas. In Zanzibar, CHVs could play a role in reaching additional high-risk groups. A study on human behaviour and residual malaria transmission in Zanzibar reported seasonal workers and migrants, generally adult males, tend to be at higher risk for malaria transmission and often have less access to malaria prevention programmes, including ITN distribution programmes and community-level education on malaria prevention. This study recommended expansion of community health programmes to reach these higher risk groups [9].

These findings also suggest CHVs need to be accepted and respected in the communities where they work, consistent with past research that has shown living in and being accepted in the community is the most basic criterion for CHVs to be approved by the community [31]. Other studies also documented that selection of volunteers should be informed by the social and cultural context in the programme area to ensure volunteers are acceptable to the community [14, 31, 32]. This has also been emphasized in the criteria for selecting the CHVs in the Zanzibar community health strategy.

As it has been practiced in this study, participants also emphasized that CHVs should be connected to community leaders and health workers in their area, noting community members will be more confident and more likely to accept door-to-door interventions if CHVs are introduced by the community leaders and are viewed to work with health care providers. Previous research suggests that community leaders are among key stakeholders to include in the design and implementation of the community engagement process and emphasized that programmes are likely to fail if local leadership does not support the programme’s goals [33].

CHVs also provided valuable insights and recommendations on how to make door-to-door interventions effective. CHVs emphasized that they need to be provided with adequate education and resources, this was mentioned as one of the most important criteria that will enable them to conduct this intervention effectively. Apart from the initial training they encourage ongoing on the job training and supportive supervision in the first few weeks and more job aids such as booklets or leaflets, to make it easy for them to provide health education sessions. Various studies have concluded that proper training of CHVs is an important step in effectively addressing diseases. One systematic review emphasized that training large numbers of CHWs can improve the detection and management of Buruli ulcer in sub-Saharan Africa [11, 32].

CHVs also emphasized that to sustainably do the work, they should receive additional payment that will enable them to meet their basic needs. The new WHO guideline on CHW programme support also recommends a financial package for CHWs [34], yet others argue that offering financial incentives is not always an effective or desirable strategy, as this may undermine the volunteering spirit [35, 36]. Some other studies support both financial and non-financial incentives, these studies documented that both financial and non-financial incentives, independently and together, improve CHW motivation [37–40]. It is critical that CHVs are able to meet their basic needs, whether through direct payment for their work, or by ensuring the time commitment required to fulfill their CHV responsibilities leaves sufficient time to do other income-generating activities. The community health strategy in Zanzibar recommends that the CHVs maximum stipend achievable should reflect the expected workload of the CHVs service package and should be reviewed periodically and adjusted as CHVs service package and responsibilities change to avoid CHV burnout and ensure CHVs serve their community effectively [21].

One study conducted in rural Kilifi in Kenya concluded that CHVs may find it difficult to strike a balance between their work, the need for economic survival, child care and community functions [41]. Strategies to support the livelihoods of CHVs through context relevant income generating activities should be identified and co-developed by the Ministry of Health and other stakeholders in consultation with the CHVs [41].

Community engagement is key for malaria elimination; a door-to-door approach has been accepted by the community members and could be one intervention to increase community engagement.

To ensure sustainability of this approach, door to door interventions needs to be streamlined in the existing policy and the national health programmes, government should allocate special funds to support community health volunteers as many projects end after a certain period of time. Regular analysis of the challenges faced by CHVs can inform programmatic decisions to avoid turnover of the CHVs.

## Study limitations

Data collection was done by trained researcher assistants’ members of the District Health Management teams. Their positions within the district health management teams i.e., government positions may have influenced how freely people were willing to speak about challenges or may have increased social desirability bias. To minimize this bias the participants were encouraged to speak freely and have an open discussion of challenges.

Due to limited resources and wanting to gather a range of perspectives from stakeholders, CHVs were not involved in IDIs and were only involved in one FGD, additional research with this group could be helpful.

While this study provided valuable perceptions of the door-to-door intervention, it did not measure its impact. Additional research is needed to better understand behavioural outcomes associated with door-to-door education. Further, qualitative research has different objectives from quantitative research and is not designed to ensure representativeness or generalizability. Perspectives of people involved in this study may be different from other stakeholders involved in the programme. However, the qualitative information collected through IDIs and FGDs provided rich insights on the implementation of the intervention and opportunities for improvement. Further, inclusion of different types of stakeholders and collection of information until saturation was reached on the topics of interest helped to ensure a range of perspectives were represented and thoroughly explored.

## Conclusion

The door-to-door intervention was perceived as helpful for promoting community engagement and has the potential to increase uptake of malaria prevention interventions and accelerate efforts towards malaria elimination. There are a number of factors to consider including addressing gender issues, ensuring that CHVs are provided with adequate education, regular supportive supervision, and have access to essential resources. Community leaders should be fully involved in choosing CHVs that are acceptable to the community and who will be respectful of cultural norms. To ensure sustainability, the government should allocate sufficient resources and improve coordination systems. This includes providing a comprehensive plan outlining roles of health service providers at different levels to increase engagement in the planning and implementation of malaria interventions.

## Data Availability

Data from this study can be accessed upon request through the corresponding author who will submit a formal request to the Ministry of Health through the Zanzibar Malaria Elimination Programme.

## Abbreviations

WHO: World Health Organization
IRS: Indoor residual spraying
SMC: Seasonal Malaria Chemoprevention
LLIN: Long-lasting insecticidal nets
CHV: Community Health Volunteers
ZAMEP: Zanzibar Malaria Elimination Programme
DMSO: District Malaria Surveillance Officers
